# Case Report: Linezolid-associated toxic optic neuropathy in patients with pulmonary tuberculosis: case series and mini review

**DOI:** 10.3389/fphar.2025.1547236

**Published:** 2025-05-13

**Authors:** Hee Jin Yun, Su-Jin Kim, Kwang Eon Han, Eun Jung Choi, Ji-Eun Lee

**Affiliations:** ^1^ Department of Ophthalmology, Pusan National University Yangsan Hospital, Pusan National University School of Medicine, Yangsan, Republic of Korea; ^2^ Research Institute for Convergence of Biomedical Science and Technology, Pusan National University Yangsan Hospital, Yangsan, Republic of Korea

**Keywords:** case report, extensive drug resistance, linezolid, muti-drug registance, toxic optic neuropathy, tuberculosis

## Abstract

Linezolid is a useful second-line medication for the treatment of tuberculosis that is resistant to multiple drugs. However, long-term therapy can cause side effects, such as optic neuropathy. We describe three cases of progressive painless bilateral visual loss in patients taking long-term linezolid therapy for multidrug-resistant and extensively drug-resistant tuberculosis. We reviewed the literatures and identified 58 cases of linezolid optic neuropathy. Discontinuation of linezolid resulted in a marked improvement in vision, however it was not complete in all patients. Knowledge of visual monitoring of patients undergoing long-term linezolid therapy is important for physicians and ophthalmologists.

## 1 Introduction

Tuberculosis (TB) is a communicable disease that contributes significantly to global mortality and morbidity and affects 10 million people worldwide annually (World Health Organization, 2018). Resistance to classical first-line anti-TB medications is common in developing and economically poor countries. The global incidence of multi-drug-resistant (MDR) *Mycobacterium tuberculosis* (TB) is 3.4% and 18% in new and previously treated patients, respectively (Yang et al., 2014). Therefore, second-line anti-TB drugs are utilized frequently used to treat MDR- and extensively drug-resistant (XDR)-TB. Linezolid is a second-line anti-TB medication approved by the World Health Organization for MDR-TB treatment (Singh et al., 2019; World Health Organization, 2014). It was originally developed to address the worsening antimicrobial resistance, particularly, of vancomycin-resistant *Enterococcus spp*. (VRE) and methicillin-resistant *Staphylococcus aureus (*MRSA) (Thwaites and Nguyen, 2022).

Linezolid is the first agent in a new class of synthetic antimicrobials, called oxazolidinones, and is a bacteriostatic antibiotic that inhibits bacterial ribosomal protein synthesis by binding to the 23S ribosomal RNA of the 50S subunit, thereby preventing the development of the 70S RNA initiation complex (Hashemian et al., 2018).

Although linezolid has demonstrated potential efficacy, its long-term use has been associated with serious adverse effects, such as optic neuropathy, myelosuppression, and peripheral neuropathies, which are especially important in patients with drug-resistant infections who require prolonged treatment (Pilania et al., 2018; Lee et al., 2012). Herein, we present three cases of patients treated with linezolid for MDR and XDR-TB with bilateral optic neuropathy. Additionally, we identified 58 cases of linezolid-associated optic neuropathy (LON) in the previous reports and summarized the cases.

## 2 Case description

### 2.1 Patient 1

A 44 -year -old woman visited the emergency department with pain in both feet and weakness in the leg. She had been treated for pulmonary TB two times, 18 and 30 years previously. Multiple cavity consolidations were found upon chest computed tomography, and the sputum test was revealed MDR-TB. TB vasculitis and spinal cord infarction were suspected based on the spine magnetic resonance images. She did not have diabetes, hypertension, kidney disease, or a history of alcohol or drug abuse. The patient restarted TB medication, including linezolid 600 mg taken once per day.

**FIGURE 1:**
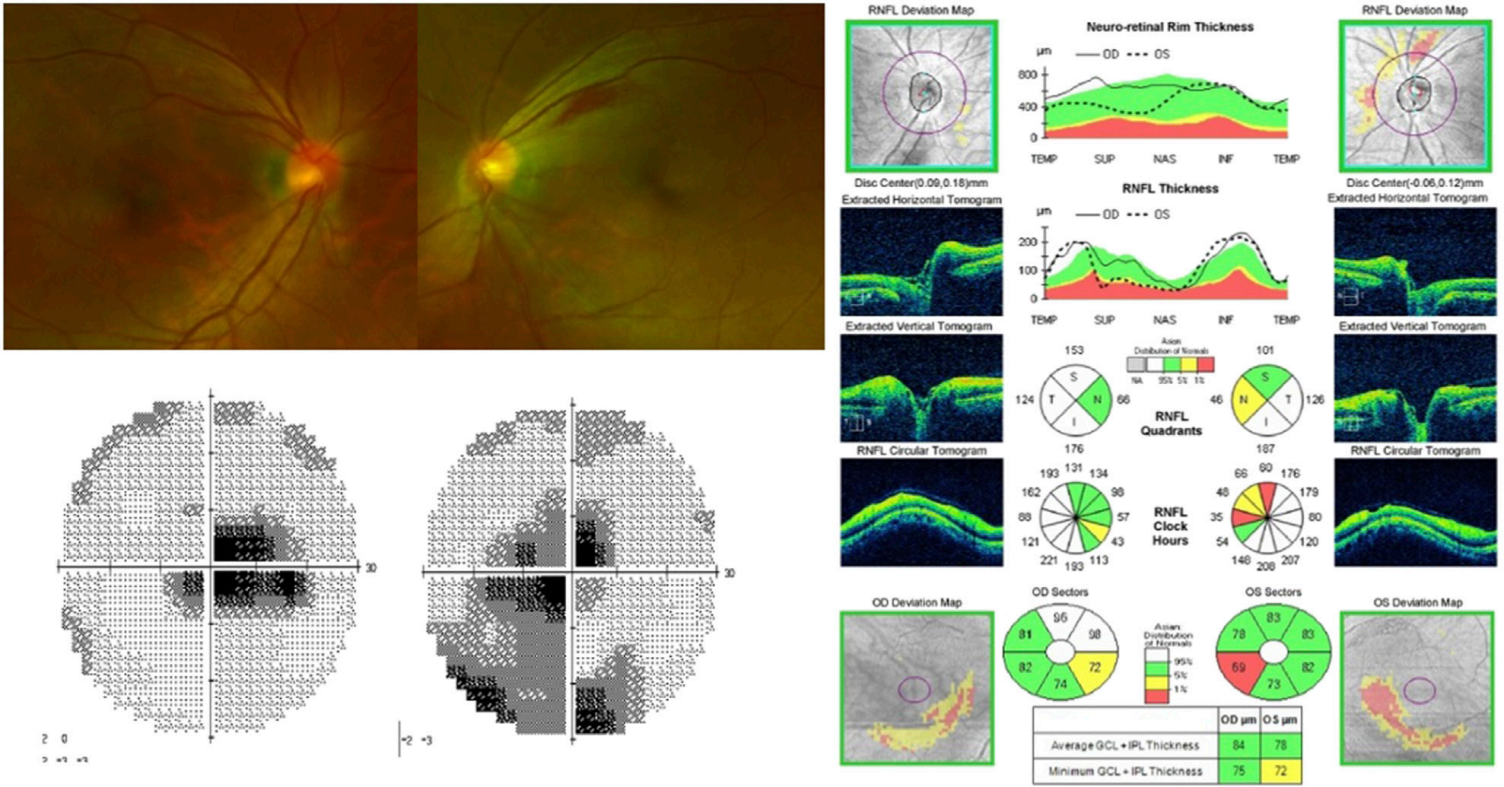
Visual field test shows central scotoma in both eyes. Fundus photography showing superficial retinal hemorrhage along the superior vascular arcade in the left eye. Optical coherence tomography reveals peripapillary retinal nerve fiber layers thickening in both eyes and thinning of macular ganglion cell-inner plexiform layer in both eyes, especially the inferonasal macular area.

After receiving linezolid therapy for 7 months, the patient complained of bilateral blurred vision. The patient was referred to the ophthalmology clinic. Her best-corrected visual acuity (BCVA) was 20/200 and “counting fingers” in the right and left eyes, respectively. She did not respond to colour vision tests in either eye, and a visual field test showed central scotoma in both eyes. Dilated fundus examination revealed superficial retinal haemorrhage along the superior vascular arcade in the left eye. Optical coherence tomography (OCT) revealed mild peripapillary retinal nerve fibre layer (RNFL) thickening and macular ganglion cell-inner plexiform layer (GC-IPL) thinning in both eyes (Figure 1).

**FIGURE 2:**
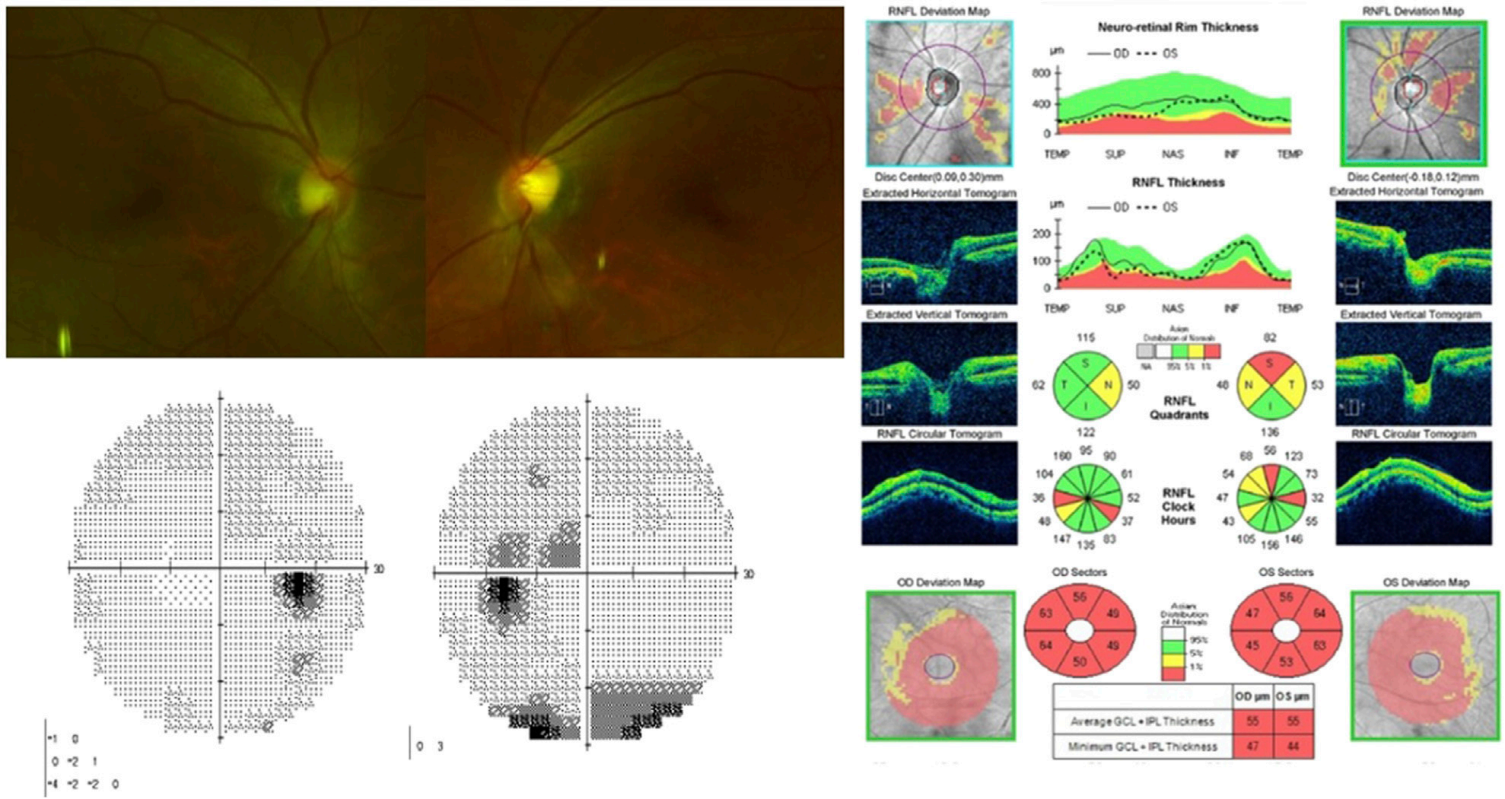
On fundus photography, superficial retinal hemorrhage in the left eye disappeared. Visual field test shows improvement of central scotoma in the right eye and partial improvement in the left eye. Optical coherence tomography indicats thinning of peripapillary retinal nerve fiber layers and progressed thinning of macular ganglion cell-inner plexiform layer.

The linezolid therapy was immediately discontinued. Two months after linezolid discontinuation, the BCVA of the patient improved to 20/20 and 12/20 in the right and left eyes, respectively. On repeat fundus examination, the retinal haemorrhage disappeared, the central scotoma in the right eye improved, and the patient regained some colour vision in both eyes; however, the central scotoma in the left eye partially remained. The OCT revealed mild thinning of the peripapillary RNFL and progressive thinning of the macular GC-IPL (Figure 2). Four months after the discontinuation of the drug, her visual acuity in the left eye improved to 20/20. Six months after discontinuation, the central scotoma in the left eye improved completely.

### 2.2 Patient 2

A 48-year-old woman with XDR-TB was treated with linezolid 600 mg daily. She did not have diabetes, hypertension, kidney disease, or a history of alcohol or drug abuse. After receiving linezolid for 10 months, the patient presented with blurred vision. Eye examination revealed that the BCVA was 10/20 and 4/20 in the right and left eyes, respectively. Colour vision was impaired in both eyes, and a visual field test revealed cecocentral scotoma in both eyes. Dilated fundus examination did not reveal any abnormalities on the disc or retina. OCT revealed mild thinning of the peripapillary RNFL and macular GC-IPL in both eyes. Four weeks after the discontinuation of the drug, the BCVA improved to 20/20 and 16/20 in the right and left eyes, respectively. The cecocentral scotoma improved completely in the right eye, and the remaining visual field defect in the left eye improved 2 months after the discontinuation.

### 2.3 Patient 3

A 71-year-old man was diagnosed with MDR-TB based on a sputum test while undergoing inpatient treatment for a brain abscess caused by *Streptococcus constellatus*. He had been treated for TB 8 years previously, and had undergone left upper lobectomy 7 years previously. He did not have diabetes, hypertension, kidney disease, or a history of alcohol or drug abuse. TB medication was started, including linezolid 600 mg once per day. Approximately 14 months after the initiation of linezolid therapy, the patient presented with blurred vision. An eye examination revealed that the BCVA was 2/200 and 4/200 in the right and left eyes, respectively. Dilated fundus examination did not reveal any abnormal findings on the disc and retina. A visual field test revealed central scotoma and blind spot enlargement in both eyes. OCT indicated the normal RNFL thickness and macular GC-IPL thinning. Three months after the discontinuation of the drug, the BCVA improved to 20/100 and 20/32 in the right and left eyes, respectively. The central scotoma partially improved in both eyes.

## 3 Methods

Three cases of linezolid-associated toxic optic neuropathy reported in our institution from 2010 to 2023 were available for review. A retrospective chart review was then performed to analyse the clinical data of these cases. To further analyse this topic, a systematic literature search was performed using two separate databases: PubMed/MEDLINE and Google Scholar. All available case reports and case series were reviewed, and the data are summarised in Table 1.

**TABLE 1:** Demographic and clinical characteristics of the patients in the previous reports.

Clinical characteristics	Value
Number of patients	58
Sex (male/female)	31/27
Age (years), mean ± SD (range)	42.6 ± 21.2 (6–81)
Daily dose (mg)	
1,600	1 (1.7)
1,200	17 (29.3)
600	38 (65.5)
Usage duration (months)	9.2 ± 8.0
Underlying disease, n (%)	
Prosthesis infection	9 (15.5)
Wound infection	3 (5.2)
Osteomyelitis	5 (8.6)
Tuberculosis	35 (60.3)
Pneumonia	5 (8.6)
Pyoderma gangarenosum	1 (1.7)
Type of bacteria, n (%)	
MRSA	18 (31.0)
MDR Tb	15 (25.9)
XDR Tb	14 (24.1)
MRSE	1 (1.7)
VRE	2 (3.4)
*Mycobacterium avium*	2 (3.4)
*Mycobacterium* bovis	2 (3.4)
*Mycobacterium* abscessus	1 (1.7)
*Mycobacterium* nonchromogeniucum	1 (1.7)
BCVA (logMAR) at first visit	
Rt. eye	1.25 ± 0.64
Lt. eye	1.12 ± 0.60
Type of Visual field defect, n (eyes)	
Cecocentral scotoma	22 (19.0)
Central scotoma	26 (22.4)
Peripheral contstriction	9 (7.8)
Undetermined	59 (50.9)
Fundus, n (eyes)	
Disc swelling	36 (31.0)
Peripapillary hemorrahge	2 (1.7)
Disc pallor	11 (9.5)
Normal	18 (15.5)
Undetermined	49 (42.2)
BCVA (logMAR) at final visit	
Rt. eye	0.23 ± 0.41
Lt. eye	0.26 ± 0.51
Time to improvement of vision (weeks)	8.79 ± 4.89
Number of eyes with no improvement	6 (10.3)

Values are presented as mean ± standard deviation, number, or number (%) unless otherwise indicated.

BCVA, best-corrected visual acuity; logMAR, logarithm of the minimum angle of resolution; MD, mean deviation; PA, pituitary adenoma; PSD, pattern standard deviation; RNFL, retinal nerve fiber layer; VFD, visual field defect; VFI, visual field index.

The study protocol adhered to the tenets of the Declaration of Helsinki and was approved by the Institutional Review Board of Pusan National University Yangsan Hospital (IRB number: 55-2024-050). Because this was a retrospective study, the need for informed consent was waived by the IRB of Pusan National University Yangsan Hospital.

## 4 Results

The search yielded 48 articles, all of which were case reports published between 2002 and 2024 (De Vriese et al., 2006; Rana et al., 2018; Vallabhaneni et al., 2024; Pilania et al., 2018; Lee et al., 2012; Mehta et al., 2016; Miller et al., 2023; Toolan et al., 2023; Brandariz-Núñez et al., 2019; Dempsey et al., 2018; Rucker et al., 2006; Azamfirei et al., 2007; Joshi et al., 2009; Saijo et al., 2005; Lee et al., 2018a; Aljebreen et al., 2020; Karuppannasamy et al., 2014; Grohmann et al., 2020; Khadilkar et al., 2013; Gonzalez et al., 2017; Libershteyn, 2016; Javaheri et al., 2007; Han et al., 2023; Bano et al., 2022; Lee et al., 2003; McKinley and Foroozan, 2005; Frippiat et al., 2004; Kulkarni and Del Priore, 2005; Kiuchi et al., 2009; Patel and Ramchandani, 2015; Agrawal et al., 2015; Park et al., 2015; Ishii et al., 2016; Han et al., 2013; Corallo and Paull, 2002; Srivastava et al., 2017; Kreps et al., 2017; Agashe and Doshi, 2019; Rosenzweig et al., 2006; Zivkovic and Lacomis, 2005; Rho et al., 2004; Shah and Lamichhane, 2017; Kardani et al., 2021; Yoon and Lee, 2019; Bhonsale and Amberkar, 2021; Berkovitz et al., 2017; Xerri et al., 2015). These case reports included 58 patients, all of whom were diagnosed with linezolid-associated toxic optic neuropathy. The number of male and female patients was 31 and 27, respectively. The patients’ mean age was 42.6 ± 21.2 (range: 6∼81) years. The daily dose of linezolid was 800 mg two times per day in one patient, 600 mg two times per day in 17 patients, 600 mg in 38 patients, and 10 mg/kg in 2 paediatric patients. The time from the initiation of medication to the appearance of visual symptoms was 9.2 ± 8.0 months. The causative diseases for which linezolid treatment was administered were prosthesis infections (n = 9), wound infections (n = 3), osteomyelitis (n = 5), TB (n = 35), pneumonia (n = 5), and pyoderma gangrenosum (n = 1). The causative organisms were MRSA (n = 18), MDR-TB (n = 15), XDR-TB (n = 14), MRSE (methicillin-resistant *Staphylococcus epidermidis*) (n = 1), VRE (n = 2), *Mycobacterium avium* (n = 2), *Mycobacterium bovis* (n = 2), *Mycobacterium abscessus* (n = 1), and *Mycobacterium nonchromogenicum* (n = 1). The BCVA at the first ophthalmic examination was 1.25 ± 0.64 logarithm of the minumun angle of resolution (logMAR) in the right eye and 1.12 ± 0.60 logMAR in the left eye. Visual field defects observed included cecocentral scotoma (n = 22), central scotoma (n = 26), and peripheral constriction (n = 9). Fundus examination revealed disc swelling (n = 36), peripapillary haemorrhage (n = 2), disc pallor (n = 11), and normal disc (n = 18). The time from discontinuation of the drug to improvement in vision was 8.79 ± 4.89 weeks. The final BCVA was 0.23 ± 0.41 in the right eye and 0.26 ± 0.51 in the left eye. Six (10.3%) eyes showed no improvement in vision.

## 5 Discussion

Our study highlights the risk of optic neuropathy during linezolid treatment, as shown in our three case presentations. Moreover, our literature search resulted in 48 previously published cases whereby the clinical picture was summarized for all cases.

The most relevant risk factor for linezolid-induced neurotoxicity is the extended duration of treatment. The Food and Drug Administration (FDA) recommends linezolid treatment for a maximum of 28 days because of its safety profile (Miller et al., 2023). Long-term use outside the 28-day window has been associated with optic and peripheral neuropathies (Sotgiu et al., 2012; Zhang et al., 2015; Agyeman and Ofori-Asenso, 2016). Zhang et al. determined that neurotoxicity with peripheral neuropathy occurs in 30% of patients who had received doses 600 mg/day or more for 4–6 months (Zhang et al., 2015). In our study, patients were administered a dose of 600 mg/day for 7–14 months before symptom onset. In the 48 previous reports, 1 patient received 1,600 mg per day, 17 adult patients received 1,200 mg per day, and 38 adult patients received 600 mg per day of the drug. We found that the time to symptom presentation was longer than that previously reported, with an average presentation time of approximately 9.2 months. Among them, three patients developed optic neuropathy quickly after less than 4 weeks of linezolid use, which is known to be a safe period.

Although the exact mechanism underlying its toxicity remains unknown, it is believed that linezolid affects the optic nerve by interfering with mitochondrial oxidative phosphorylation. The combination of energy deficiency and oxidative stress leads to cytochrome C leakage from mitochondrial pores, causing apoptosis and nerve damage (Han et al., 2023; Garrabou et al., 2017; McKee et al., 2006). The macula is an area packed with photoreceptors whose axons form the most important bundle of fibres, the papillomacular bundle, which enters the optic nerve. The mitochondria-rich papillomacular bundle is mainly damaged by toxic medications, and appears as a cecocentral or central scotoma in the visual field test. Among the cases in which a visual field defect was reported (n = 57), 84.2% (48/57) had a cecocentral or central scotoma. The optic disc may be normal or slightly hyperaemic during early detection and temporal optic disc pallor may be observed during late detection. We found optic disc oedema in 53.7% (36/67) of patients, among the cases in which a fundus examination was reported (n = 67). Temporal RNFL and macular GC-IPL thicknesses are decreased in toxic optic neuropathy, reflecting damage to the papillomacular bundle. In previous studies, OCT findings were reported in 16 cases of 58 patients, and 12 of 16 patients showed RNFL thickening. In our cases, all the three patients revealed central or cecocentral scotoma on visual field test, no one had disc swelling or pale, and OCT indicated thinning of the macular GC-IPL, but variable RNFL thickness. This is because the initial RNFL swelling occurred within 3 months of symptom onset and then decreased over time (Lee et al., 2018b).

A clinical trial by Pfizer reported an incidence of optic neuropathy in 4.17% of patients taking linezolid for any indication. Several studies have reported that the incidence of optic neuropathy in patients with drug-resistant TB is much higher (8∼19%) (Zhang et al., 2015; Pfizer and Inc, 2015). One possible explanation for the higher incidence of LON in patients with MDR or XDR-TB is that these patients are commonly systemically ill with pulmonary or renal dysfunction, which may predispose them to toxicity (Miller et al., 2023). Among the 58 patients form our review of literature, 60.3% (35/58) had tuberculosis, and 50% had MDR- or XDR-TB. In our cases, all the three patients took linezolid for treatment of MDR or XDR TB.

Currently, no treatments are available for LON. Linezolid should be discontinued immediately in patients with visual disturbances suspected to be optic neuropathy (Thwaites and Nguyen, 2022; Lee et al., 2018c). Nutritional supplementation, including the vitamins thiamine (B1), riboflavin (B2), niacin (B3), pyridoxine (B6), cobalamin (B12), folic acid, and proteins with sulfur-containing amino acids, could serve as potential supportive treatments (Sharma and Sharma, 2011). When the medication is withdrawn, most patients with optic nerve injury improve, likely because the triggering factor is removed before apoptosis and permanent axonal loss occur (Garrabou et al., 2017; McKee et al., 2006). Previous reviews have reported that 82%–92.3% of patients experience complete resolution of visual disturbances (Brandariz-Núñez et al., 2019; Dempsey et al., 2018). Among the 58 patients from our review of literature, six (10.3%) showed no improvement in visual acuity. Among our three patients, the visual impairment in one patient did not resolve entirely even after 3 months. However, the exact duration of symptom relief following drug withdrawal remains unclear. In previous reports, optic neuropathy resolved within a mean of 8.8 weeks after drug discontinuation. Among our patients, two patients showed complete visual recovery 2 months after drug withdrawal.

The occurrence and risk factors of linezolid intoxication have not been thoroughly investigated. Several researchers have hypothesised that patients with pre-existing neurological sequelae or risk factors such as alcoholism, diabetes, or concurrent use of chemotherapeutic drugs are more susceptible to linezolid-induced neurotoxicity (Lee et al., 2012). The currently recommended linezolid dose is typically 600 mg one or two times daily (Thwaites and Nguyen, 2022). The correlation between the dosage and the occurrence of optic neuropathy has not yet been clearly identified. Miller et al. reported that patients receiving a dose of 600 mg two times daily experienced a much lower rate of full recovery (44.4%) than did those receiving 300 mg daily (66.7%) and those receiving 600 mg daily (71.4%), suggesting that a higher dose may carry a higher risk of optic nerve toxicity, and increase the likelihood of permanent visual impairment (Miller et al., 2023). Among our patients, the total dosage administered to the third patient was higher than that administered to the other two patients (600 mg daily for 14 months), he was older and his general condition was poor, including a brain abscess and a history of lung resection, which appeared to have contributed to his incomplete vision recovery.

The use of systemic corticosteroids for treating LON is controversial with some authors suggesting that corticosteroids contribute to worse outcomes (Azamfirei et al., 2007; Saijo et al., 2005). In one case from the previous reports, the patient begun receiving oral corticosteroids to treat optic neuropathy experienced worsening of their visual acuity and fields (Saijo et al., 2005). In contrast, in their report, Mehta et al. described three patients treated with oral corticosteroids whose visual acuity improved to the baseline level (Mehta et al., 2016). None of our three patients received steroid treatment, and two of them showed visual recovery after discontinuation of linezolid.

Several studies have proposed formal screening guidelines for patients receiving linezolid for a long term. Dempsey et al. recommended a baseline examination at 1 month, followed by repeated examinations every 30–60 days during treatment (Dempsey et al., 2018). Baseline testing followed by regular fundus examinations with automated perimetry, colour vision testing, and OCT may help patients avoid permanent optic nerve injury or vision loss. However, further research is required to identify the best methods for optic nerve toxicity monitoring.

## 6 Conclusion

Regular monitoring is essential for patients with long-term linezolid use. If there is painless, bilateral, and progressive visual impairment with dyschromatopsia, central/cecocentral visual field defects, or papillomacular bundle defects, toxic optic neuropathy should be suspected, and immediate ophthalmologic evaluation and discontinuation of medication are mandatory. Additional research is required to determine the parameters associated with visual prognosis.

## Data Availability

The original contributions presented in the study are included in the article/Supplementary Material, further inquiries can be directed to the corresponding author.
